# Proposal of two novel species, Allocoprococcus similis gen. nov., sp. nov. and Faecalimonas hominis sp. nov., isolated from human faeces and genome-based reorganization of the genus Coprococcus

**DOI:** 10.1099/ijsem.0.007011

**Published:** 2026-01-07

**Authors:** Eri Yamamoto, Atsushi Hisatomi, Kana Miwa, Naomi Sakurai, Akiko Koizumi, Moriya Ohkuma, Hanae Tsuchihashi, Mitsuo Sakamoto

**Affiliations:** 1 Microbial Bioresources Group, Lactic Acid Bacteria & Fermentation Technology Research Unit, R&D Division, Meiji Co., Ltd., Hachioji, Tokyo 192-0919, Japan; 2 Microbe Division/Japan Collection of Microorganisms, RIKEN BioResource Research Center, Tsukuba, Ibaraki 305-0074, Japan; 3 NODAI Culture Collection Center, Tokyo NODAI Research Institute, Tokyo University of Agriculture, Setagaya-ku, Tokyo 156-8502, Japan

**Keywords:** *Allocoprococcus*, *Coprococcus*, *Faecalimonas*, genome-based taxonomy, human faeces, *Pseudocoprococcus*

## Abstract

The obligately anaerobic, Gram-stain-positive coccobacilli strains OB7620^T^ and OB7656^T^ were isolated from faecal samples of healthy Japanese volunteers. Strain OB7620^T^ showed the highest 16S rRNA gene sequence similarity to *Coprococcus comes* ATCC 27758^T^ (98.4%) and *Clostridium nexile* DSM 1787^T^ (96.3%). Strain OB7656^T^ showed the highest 16S rRNA gene sequence similarity to ‘*Gluceribacter canis*’ NATH-2371^T^ (97.7%), *C. nexile* DSM 1787^T^ (94.8%) and *Faecalimonas umbilicata* EGH7^T^ (94.8%). These findings indicate that the two strains represent novel species. Genomic analysis clarified the phylogenetic relationship of *C. comes* with the species cluster designated as *Coprococcus sensu stricto*, which includes the type species, *C. eutactus*. As *C. comes* is distinct from the species of *Coprococcus sensu stricto*, *C. comes* is reclassified into a novel genus, *Allocoprococcus*, as *Allocoprococcus comes* gen. nov., comb. nov. Although strain OB7620^T^ is closely related to *C. comes*, it is a distinct species. Therefore, we propose the name *Allocoprococcus similis* gen. nov., sp. nov. The type strain is OB7620^T^ (=DSM 118890^T^=JCM 37173^T^). Because *Coprococcus catus* differs from *Coprococcus sensu stricto*, it has been placed in a novel genus, *Pseudocoprococcus*, as *Pseudocoprococcus catus* gen. nov., comb. nov. We also concluded that *Coprococcus immobilis* and *Coprococcus intestinihominis* are the same species and belong to the new genus *Pseudocoprococcus*. As *C. immobilis* has priority, we propose the name *Pseudocoprococcus immobilis* gen. nov., comb. nov. We also concluded that *C. nexile* and *Clostridium phoceensis* are the same species in the genus *Faecalimonas*. As *C. nexile* has priority, we propose the name *Faecalimonas nexilis* comb. nov. *Coprococcus mobilis* and ‘*G. canis*’ should also be reclassified within the genus *Faecalimonas*. Therefore, we propose the name *Faecalimonas mobilis* comb. nov. and *Faecalimonas canis* sp. nov. As strain OB7656^T^ is related to ‘*G. canis*’ but is a different species, we propose the name *Faecalimonas hominis* sp. nov. The type strain is OB7656^T^ (=DSM 118889^T^=JCM 37172^T^).

## Introduction

We isolated intestinal bacteria to gain a better understanding of the human gut microbiota. Recently, we isolated strains OB7620^T^ and OB7656^T^, which are closely related to *Coprococcus comes* and *Faecalimonas umbilicata*, respectively. The genus *Coprococcus* belongs to the family *Lachnospiraceae* and comprises phylogenetically heterogeneous species. For instance, *C. comes* and *Coprococcus catus* are distantly related in phylogenetic analyses based on 16S rRNA gene sequences [1]. This indicates that the genus *Coprococcus*, as currently defined, includes species with diverse evolutionary origins, suggesting the need for taxonomic revision. The genus *Coprococcus* could be reclassified into several different genera [2]. The Rules of the *Prokaryotic Code* require that the types of all species and subspecies with new names (including new combinations) be deposited in at least two publicly accessible culture collections in different countries. However, the type strains of *C. comes* and *C. catus* are only deposited in the American Type Culture Collection (ATCC) as ATCC 27758^T^ and ATCC 27761^T^, respectively, without a second independent deposit, as required by the *Code*. Consequently, even if such a proposal is made under the current circumstances, these species will not be approved for transfer to a different genus. This is because the resulting species name would not comply with the rules governing the valid publication of species names and the deposition of type material (Rules 27 and 30) and would, therefore, not be considered a validly published name. Following continued discussions with the ATCC, three strains were deposited in the Japan Collection of Microorganisms (JCM): *C. comes* ATCC 27758^T^, *Coprococcus eutactus* ATCC 27759^T^ and *C. catus* ATCC 27761^T^, which were assigned the strain numbers JCM 37939^T^, JCM 37940^T^ and JCM 37941^T^, respectively. Therefore, the aim of this study was to reclassify some species of *Coprococcus*. The taxonomic positions of strains OB7620^T^ and OB7656^T^ were also determined.

This study addresses a critical gap in the taxonomy of the family *Lachnospiraceae*, in which the genus *Coprococcus* has long been recognized as phylogenetically heterogeneous. We provide a more natural and evolutionarily consistent framework by reclassifying species into novel genera and clarifying their relationships using genomic evidence. These taxonomic revisions will improve the accuracy of microbial identification in gut microbiome studies, facilitate comparative genomics and support clinical research linking specific taxa to human health and disease.

## Isolation and ecology

This study was approved by the Meiji Institutional Review Board (approval no. 167). Faecal samples were collected from healthy Japanese volunteers (subject no. 1: 30-year-old man; subject no. 2: 51-year-old woman) in May 2019 in Hachioji, Tokyo, Japan, with informed consent obtained from each volunteer prior to the experiment. Faecal samples (0.5 g) were suspended in 4.5 ml of distilled water, diluted ten-fold with modified Gifu anaerobic medium (GAM, Nissui) broth containing 15% glycerol, and stored at −80 °C until use. The stored faecal samples were serially diluted and plated onto glucose blood liver (BL) agar plates (Eiken Kagaku) supplemented with 5% (v/v) horse blood. Strains OB7620^T^ (from subject no. 1) and OB7656^T^ (from subject no. 2) were isolated after 2 days of incubation at 37 °C in an anaerobic chamber with a gas mixture of H_2_/CO_2_/N_2_ (1:1:8, by vol.). The strains grew under the above gas mixture but did not grow after subculture under microaerobic conditions using the AnaeroPack-MicroAero system (Mitsubishi Gas Chemical).

## 16S rRNA gene sequence determination and phylogenetic analysis

**Fig. 1.**
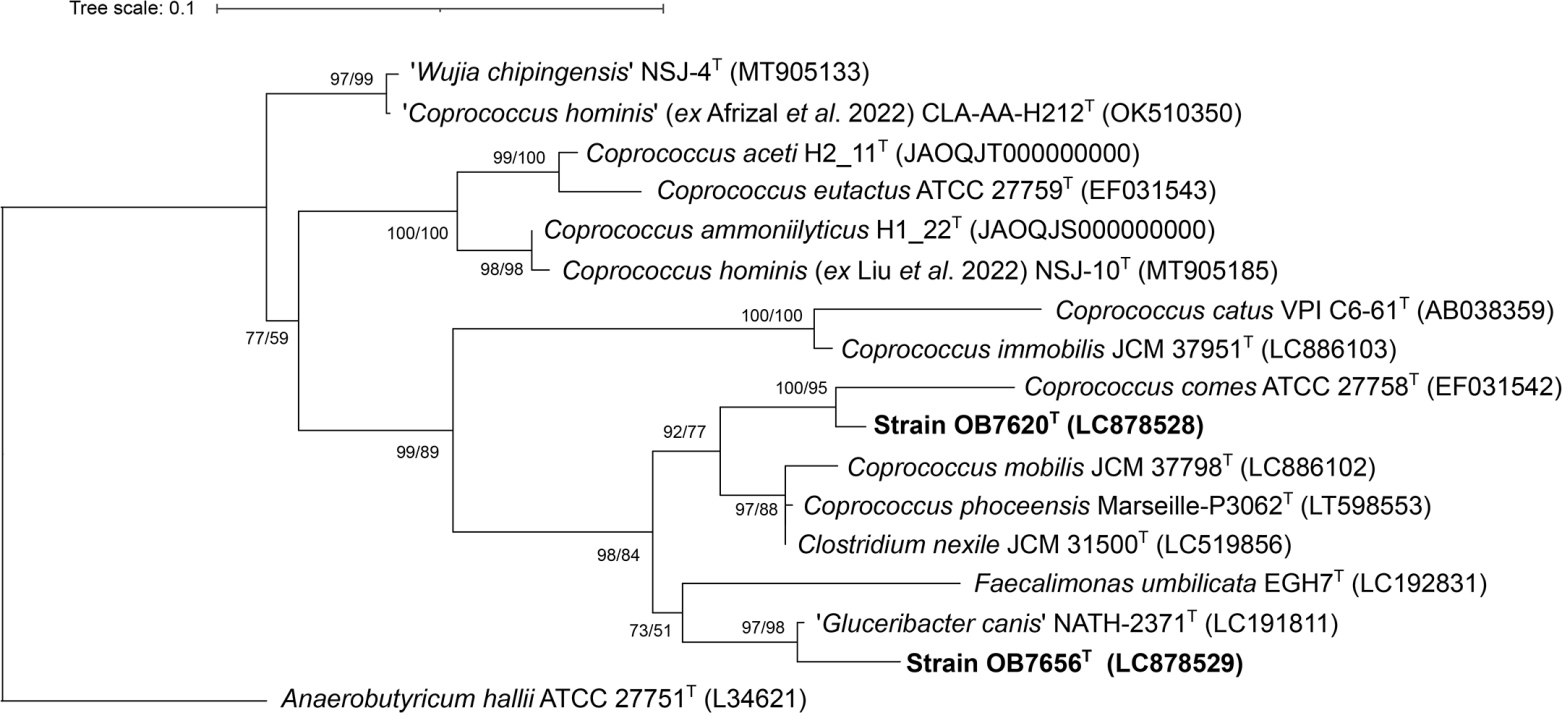
ML phylogenetic tree based on 16S rRNA gene sequences. GenBank accession numbers are indicated in parentheses. Values of 1,000 replicates for SH-aLRT (left, %) and ultrafast bootstrap (right, %) are shown at the branch nodes (>50%). *Anaerobutyricum hallii* ATCC 27751^T^ was used as an outgroup in this study.

Genomic DNA was extracted using the Wizard Genomic DNA Purification Kit (Promega). The 16S rRNA gene was analysed using the primers 27F and 1492R, as previously described [3]. A total of ~1,500 bp of 16S rRNA gene sequences were determined. Sequences were analysed by searching the GenBank/EMBL/DDBJ and EzBioCloud databases (https://www.ezbiocloud.net/identify) [4]. Strain OB7620^T^ showed the highest 16S rRNA gene sequence similarity to those of *C. comes* ATCC 27758^T^ (98.4%) and *Clostridium nexile* DSM 1787^T^ (96.3%). Strain OB7656^T^ showed the highest 16S rRNA gene sequence similarity to ‘*Gluceribacter canis*’ NATH-2371^T^ (97.7%), *C. nexile* DSM 1787^T^ (94.8%) and *F. umbilicata* EGH7^T^ (94.8%). Related sequences were retrieved from the National Center for Biotechnology Information (NCBI) database (https://www.ncbi.nlm.nih.gov/). The alignment for tree construction was generated using the sina aligner version 1.2.12 on the Silva website [5] and trimmed using trimAl version 1.3 [6] with the ‘automated1’ option. The maximum-likelihood (ML) tree was reconstructed using IQ-TREE [7] with the GTR+F+I+G model. Values of 1,000 replicates were calculated for the Shimodaira-Hasegawa-like approximate likelihood ratio test (SH-aLRT) and ultrafast bootstrap analyses. Based on 16S rRNA gene sequence analysis, strain OB7620^T^ and *C. comes* ATCC 27758^T^ were grouped into a distinct phylogenetic cluster. This cluster was clearly separated from another cluster comprising four *Coprococcus* species, among which was the type species, *C. eutactus* (Fig. 1). Strain OB7656^T^ and ‘*G. canis*’ NATH-2371^T^ formed a cluster with *F. umbilicata*.

## Genome sequence determination and phylogenetic analysis

The whole genomes of strains OB7620^T^, OB7656^T^ and ‘*G. canis*’ JCM 31739^T^ were sequenced as described below. Each strain was anaerobically cultured in GAM broth at 37 °C for 1 day. DNA was extracted using the Wizard Genomic DNA Purification Kit (Promega) and purified using the Genomic DNA Clean and Concentrator (ZYMO RESEARCH). Whole-genome sequencing of each strain was performed using an Illumina MiSeq platform. The library for Illumina MiSeq 2×300 bp paired-end sequencing was prepared using the Nextera XT DNA Sample Prep Kit (Illumina). All MiSeq reads were trimmed and assembled using the CLC Genomics Workbench version 11.0.1 (QIAGEN). Gene prediction and genome annotation of the generated contigs were performed using the DFAST pipeline (v. 1.3.6) (https://dfast.ddbj.nig.ac.jp/) [8]. The degree of genome completeness and contamination was assessed using CheckM (version 1.0.18) [9]. The genomic characteristics of the sequenced strains are listed in Table 1. Average nucleotide identity (ANI) values were calculated using the ANI calculator (https://www.ezbiocloud.net/tools/ani) [10], and digital DNA–DNA hybridization (dDDH) values were determined using the Genome-to-Genome Distance Calculator (GGDC) version 3.0 (the local alignment tool used was blast+; https://ggdc.dsmz.de/ggdc.php#) [11].

**Table 1.** Genomic features of the strains sequenced Strains: 1, OB7620^T^; 2, OB7656^T^; 3, JCM 31739^T^.

Characteristic	1	2	3
Proposed name	*A. similis*	*F. hominis*	*F. canis*
GenBank accession number	BAAHSG010000001–BAAHSG010000078	BAAHSH010000001–BAAHSH010000099	BAAHSI010000001–BAAHSI010000054
Genome size (Mbp)	3.50	2.36	2.24
No. of contig	78	99	54
N50 contig size (bp)	106,106	46,114	72,481
Largest contig size (bp)	298,707	153,499	199,540
Total genes	3,501	2,335	2,205
No. of CDS	3,426	2,274	2,153
Pseudo genes	381	267	195
G+C content (mol%)	42.6	38.9	36.8
Completeness (%)	99.4	98.8	98.8
Contamination (%)	0	0.6	0.1

CDS, coding sequence.

**Fig. 2.**
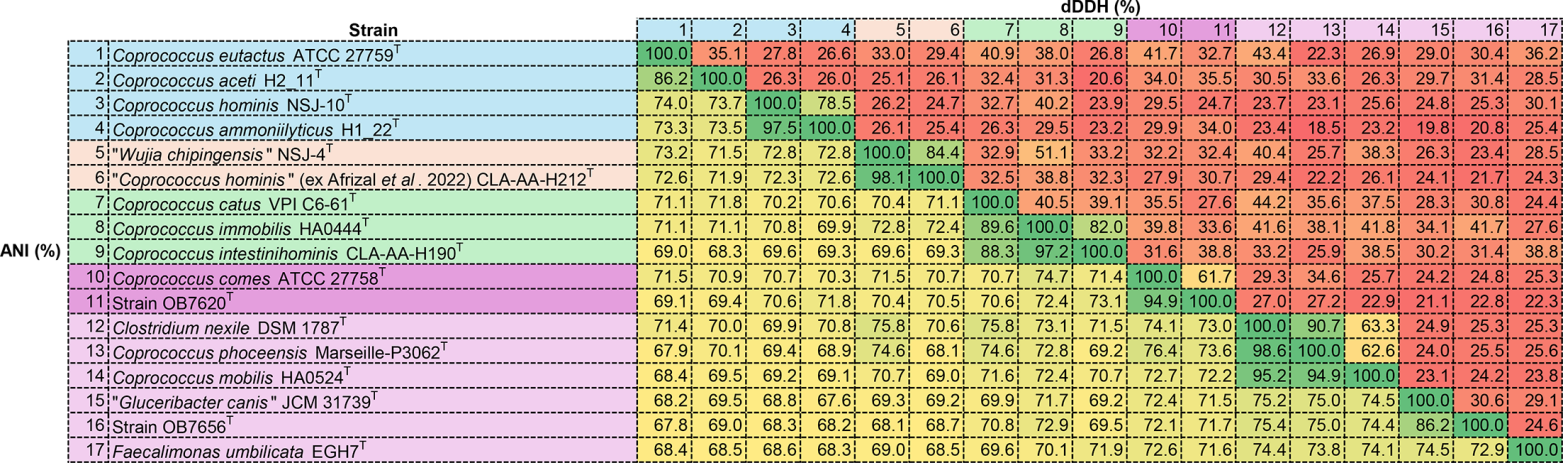
ANI (bottom) and dDDH (top) values (%) between strains OB7620^T^, OB7656^T^ and related taxa.

The ANI and dDDH results for the strains OB7620^T^, OB7656^T^, ‘*G. canis*’ JCM 31739^T^ and other taxa are summarized in Fig. 2. The ANI (94.9%) and dDDH (61.7%) values between strain OB7620^T^ and the closest type strain, *C. comes* ATCC 27758^T^, were below the proposed species cut-off of 95% [12] for ANI and 70% for dDDH, indicating that strain OB7620^T^ is a novel species. In addition, the ANI and dDDH values between strain OB7656^T^ and ‘*G. canis*’ JCM 31739^T^ were significantly lower (86.2% ANI and 30.6% dDDH) than the cut-off values for species delimitation, indicating that strain OB7656^T^ is a species different from ‘*G. canis*’.

The ANI and dDDH values between *Coprococcus ammoniilyticus* H1_22^T^ [13,14] and *Coprococcus hominis* NSJ-10^T^ (*ex* Liu *et al*. 2022) [15,16] were higher (97.5% ANI and 78.5% dDDH) than the cut-off values for species delimitation, indicating that these two strains belong to the same species. As the name *C. ammoniilyticus* Hitch *et al.* 2022 has priority over *C. hominis* Liu *et al*. 2022, the species *C. hominis* Liu *et al*. 2022 should be considered a later heterotypic synonym of *C. ammoniilyticus* Hitch *et al.* 2022. The ANI and dDDH values between *C. nexile* DSM 1787^T^ [17] and *Clostridium phoceensis* Marseille-P3062^T^ [18,19] were also higher (98.6% ANI and 90.7% dDDH) than the cut-off values for species delimitation, indicating that these two strains are the same species. As the name *C. nexile* Holdeman and Moore 1974 (Approved Lists 1980 [20]) has priority over *C. phoceensis* Bonnet *et al*. 2025, the species *C. phoceensis* Bonnet *et al*. 2025 should be considered a later heterotypic synonym of *C. nexile* Holdeman and Moore 1974 [20]. Notably, although *C. nexile* was reclassified as ‘*Tyzzerella nexilis*’ in 2013 [21], this species name has not yet been validated. In addition, the ANI and dDDH values between ‘*Wujia chipingensis*’ NSJ-4^T^ [15] and ‘*C. hominis*’ CLA-AA-H212^T^ (*ex* Afrizal *et al*. 2022) [22] were higher (98.1% ANI and 84.4% dDDH) than the cut-off values for species delimitation, indicating that these two strains belong to the same species. Afrizal *et al*. [22] have pointed this out. It has been reported that ‘*W. chipingensis*’ NSJ-4^T^ has flagella [15]. ‘*C. hominis*’ CLA-AA-H212^T^ (*ex* Afrizal *et al.* 2022) has not been reported to have flagella [22], but genome annotation results suggest that it does (data not shown). Moreover, the ANI and dDDH values between *Coprococcus immobilis* HA0444^T^ [23,24] and *Coprococcus intestinihominis* CLA-AA-H190^T^ [2,25] were higher (97.2% ANI and 82.0% dDDH) than the cut-off values for species delimitation, indicating that these two strains belong to the same species. As the name *C. immobilis* Huang *et al*. 2025 has priority over *C. intestinihominis* Hitch *et al.* 2025, the species *C. intestinihominis* Hitch *et al.* 2025 should be considered a later heterotypic synonym of *C. immobilis* Huang *et al*. 2025.

**Fig. 3.**
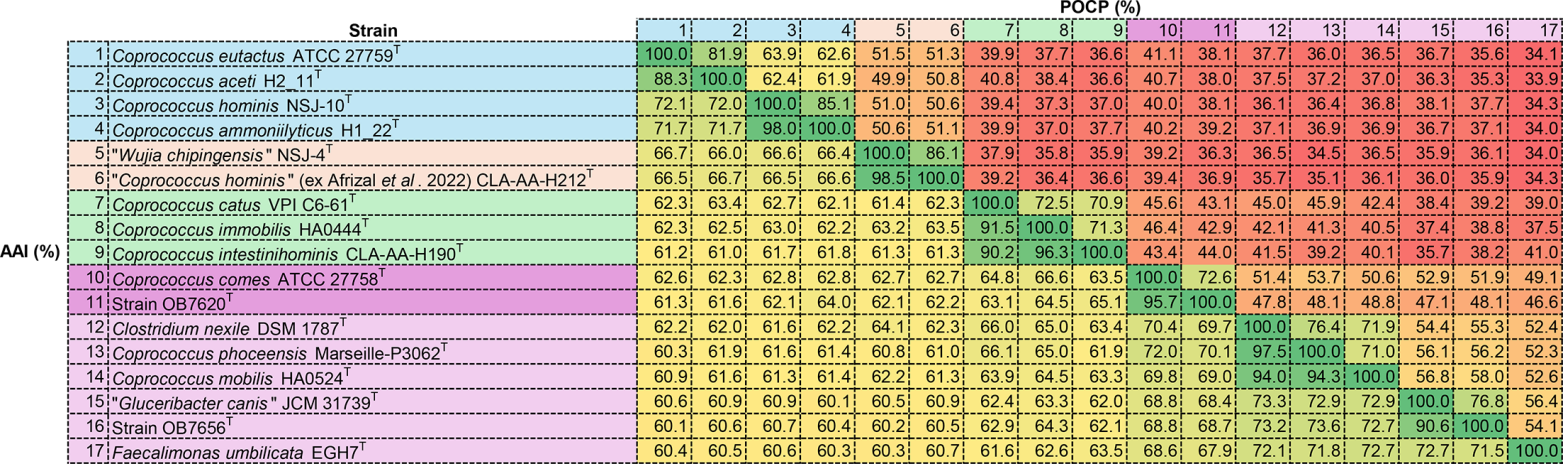
AAI (bottom) and POCP (top) values (%) between strains OB7620^T^, OB7656^T^ and related taxa.

Genus-level delineation was inferred using the percentage of conserved proteins (POCPs) [26] calculated using the blastp programme [27]. The average amino acid identity (AAI) values were calculated using EzAAI [28]. The AAI and POCP results are presented in Fig. 3. The strains analysed were divided into five groups: (I) *Coprococcus sensu stricto* (*C. eutactus* ATCC 27759^T^, *Coprococcus aceti* H2_11^T^, *C. hominis* NSJ-10^T^ and *C. ammoniilyticus* H1_22^T^); (II) the genus ‘*Wujia*’ [‘*W. chipingensis*’ NSJ-4^T^ and ‘*C. hominis*’ (*ex* Afrizal *et al.* 2022) CLA-AA-H212^T^]; (III) a novel genus, *Pseudocoprococcus* (*C. catus* VPI C6-61^T^, *C. immobilis* HA0444^T^ and *C. intestinihominis* CLA-AA-H190^T^); (IV) a novel genus, *Allocoprococcus* (*C. comes* ATCC 27758^T^ and strain OB7620^T^) and (V) the genus *Faecalimonas* (*F. umbilicata* EGH7^T^, *C. nexile* DSM 1787^T^, *C. phoceensis* Marseille-P3062^T^, *Coprococcus mobilis* HA0524^T^, ‘*G. canis*’ NATH-2371^T^ and strain OB7656^T^).

**Fig. 4.**
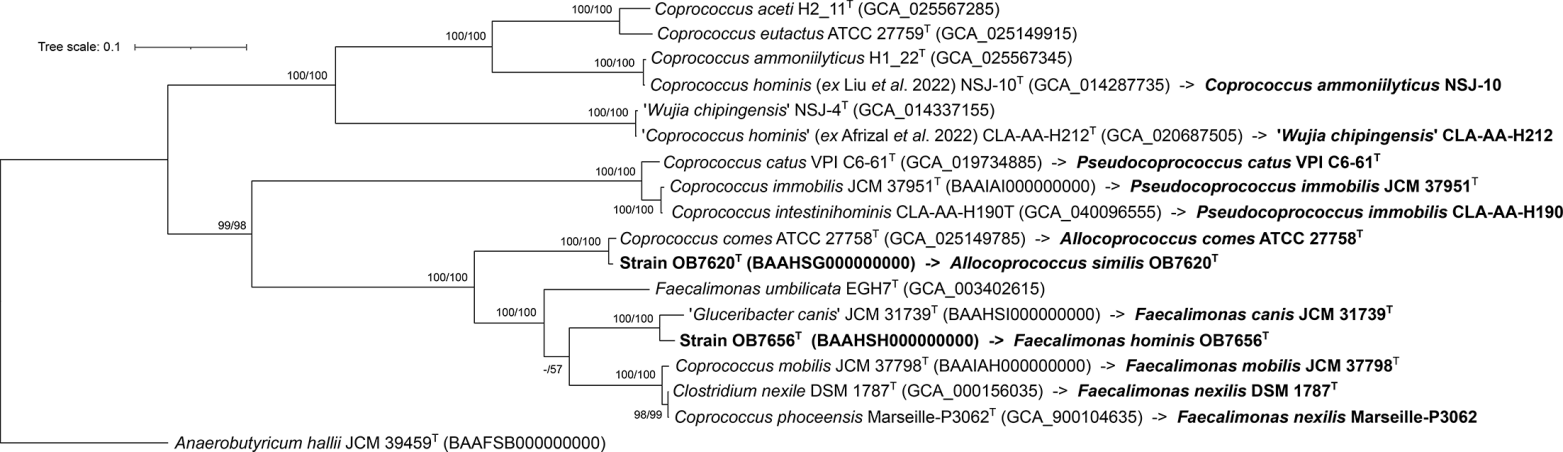
Phylogenomic tree of strains OB7620^T^, OB7656^T^ and related taxa, reconstructed using an aligned concatenated set of 120 single-copy bacterial marker proteins. The genome accession numbers are indicated in parentheses. Values of 1,000 replicates for SH-aLRT (left, %) and ultrafast bootstrap (right, %) are shown at branch nodes (>50%). *Anaerobutyricum hallii* JCM 39459^T^ was used as an outgroup.

Phylogenomic tree reconstruction was performed using 120 single-copy bacterial marker proteins. These markers were selected and aligned using GTDB-tk [29] based on the Genome Taxonomy Database (GTDB) release R220 [30]. These alignments were trimmed using TrimAl [6] with the ‘automated1’ option. ML trees for amino acid sequences were reconstructed using IQ-TREE [7] with the LG+F+I+R3 model. Values of 1,000 replicates were calculated for SH-aLRT and ultrafast bootstrap analyses. The phylogenomic tree was consistent with the relationships of each strain based on the ANI, dDDH, AAI and POCP results (Fig. 4).

## Morphology, physiology and chemotaxonomy

**Fig. 5.**
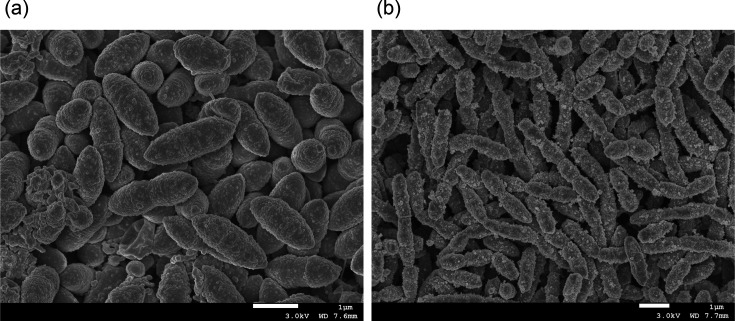
Electron micrographs of the cells of strains OB7620^T^ (**a**) and OB7656^T^ (**b**). Images were obtained using SEM.

The strains used in this study were grown on Eggerth-Gagnon (EG) agar or in GAM broth for 2–3 days at 37 °C under a gas mixture of H_2_/CO_2_/N_2_ (0.5:0.5:9, by vol.). The strains were obtained from the JCM at the RIKEN BioResource Research Center (Tsukuba, Japan). Three strains, *C. comes* ATCC 27758^T^, *C. eutactus* ATCC 27759^T^ and *C. catus* ATCC 27761^T^, were obtained from the ATCC (Virginia, USA). Cells from cultures grown on EG agar for 1 day at 37 °C under anaerobic conditions were examined by Gram staining. Cell morphology was observed using phase-contrast microscopy (BIOPHOT; Nikon). The morphologies of strains OB7620^T^ and OB7656^T^ were observed using scanning electron microscopy (SEM; JSM-6340F; JEOL). The sample preparation for SEM has been described previously [31]. Cells of strain OB7620^T^ were obligately anaerobic, non-pigmented, Gram-stain-positive coccobacilli. Cells on EG agar were 1.3–1.5×1.8–2.3 µm in size and occurred in pairs or chains. Colonies on EG agar plates were 0.7–1.2 mm in diameter, grey, circular, conical and smooth. Cells of strain OB7656^T^ were obligately anaerobic, non-pigmented, Gram-stain-positive and elongated cocci. Cells on EG agar were 0.9–1.1×2.0–2.2 µm in size and occurred in pairs and long chains. Colonies on EG agar plates were 1.4–2.4 mm in diameter, white, opaque, circular, convex, and smooth. The SEM images of the cells of strains OB7620^T^ and OB7656^T^ are shown in Fig. 5. Typical spore structures, as reported by Browne *et al*. [32], were not observed in OB7620^T^ or OB7656^T^.

The strains were incubated at different temperatures (8, 20, 25, 30, 37, 42, 45 and 50 °C) to examine bacterial growth. Strain OB7620^T^ grew at temperatures ranging from 30 to 45 °C, with optimum growth at 37 °C. In contrast, strain OB7656^T^ grew at temperatures ranging from 25 to 45 °C, with optimum growth at 37 °C. The isolates were cultivated to determine the optimum pH (pH 3.0, 4.0, 5.0, 5.5, 6.0, 6.5, 7.0, 7.5, 8.0, 8.5, 9.0 and 9.5). Strain OB7620^T^ grew at pH 5.5–8.0 (optimum pH 7.0). In contrast, strain OB7656^T^ grew at pH 5.5–8.5 (optimum pH 7.0).

The isolates were non-motile, as determined by a motility test in which tubes containing semisolid (0.15% or 0.5% agar) GAM medium were stab-inoculated and incubated at 37 °C for 2 days [33]. *C. mobilis* JCM 37798^T^, which has been reported to have motility [23], was used as a reference strain. In this study, the motility of *C. mobilis* JCM 37798^T^ was confirmed. No motility was observed in the other strains tested.

Bile resistance was tested by growing the bacteria on GAM agar plates supplemented with 0.5% or 2% (w/v) Difco-Oxgall (BD). Strains OB7620^T^ and OB7656^T^ grew in the presence of 2% Oxgall. The salinity tolerance range was tested in GAM broth with NaCl concentrations ranging from 0% to 5% (w/v) (in 1% increments). Strain OB7620^T^ grew in 1% (w/v) NaCl. In contrast, strain OB7656^T^ grew in 2% (w/v) NaCl. Catalase activity was assessed by measuring gas formation after adding fresh cells to a 3% H_2_O_2_ solution. Catalase activity was absent in all isolates. Aesculin hydrolysis was determined as described by Sakamoto *et al*. [34]. Aesculin hydrolysis was negative for OB7620^T^ and OB7656^T^. Substrate utilization was determined as described previously [34]. Biochemical reactions were performed in triplicate using the API ZYM system and Rapid ID 32A anaerobe identification kit (bioMérieux) according to the manufacturer’s instructions. The results are presented in Table S1 (available in the online Supplementary Material) and the species descriptions.

The organic acids produced as metabolic end products in GAM broth supplemented with 1% glucose (w/v) were analysed using an HPLC system (Shimadzu). The system consisted of two connected ICSep ICE ORH-801 columns (Concise Separations) and a CCD-10A electrical conductivity detector (Shimadzu). The mobile and reaction phases consisted of 7.5 mM *p*-toluene sulphonic acid monohydrate (Fujifilm Wako) in distilled water and 7.5 mM *p*-toluene sulphonic acid, 150 µM EDTA-2Na (Dojindo) and 30 mM Bis-Tris (Dojindo) in distilled water, respectively. The elution flow and column temperature were set at 0.5 ml min^−1^ and 55 °C, respectively. The end products of strain OB7620^T^ were acetate (6.73±0.56 mM), butyrate (19.61±2.12 mM), formate (2.77±0.24 mM), lactate (38.36±2.07 mM) and propionate (3.19±0.04 mM), with small amounts of pyruvate (0.08±0.03 mM). This result is consistent with that of the closely related species *C. comes* ATCC 27758^T^. In contrast, the end products of strain OB7656^T^ were acetate (16.74±0.46 mM), formate (18.71±0.54 mM), lactate (0.35±0.05 mM), propionate (1.4±0.09 mM) and pyruvate (1.77±1.26 mM). Strain OB7656^T^, *F. umbilicata* JCM 30896^T^ and ‘*G. canis*’ JCM 31739^T^ did not produce butyrate, whereas strain OB7620^T^ and *C. comes* ATCC 27758^T^, which are related to these strains, produced large amounts of butyrate (19.6 and 22.3 mM, respectively) (Table S2).

**Fig. 6.**
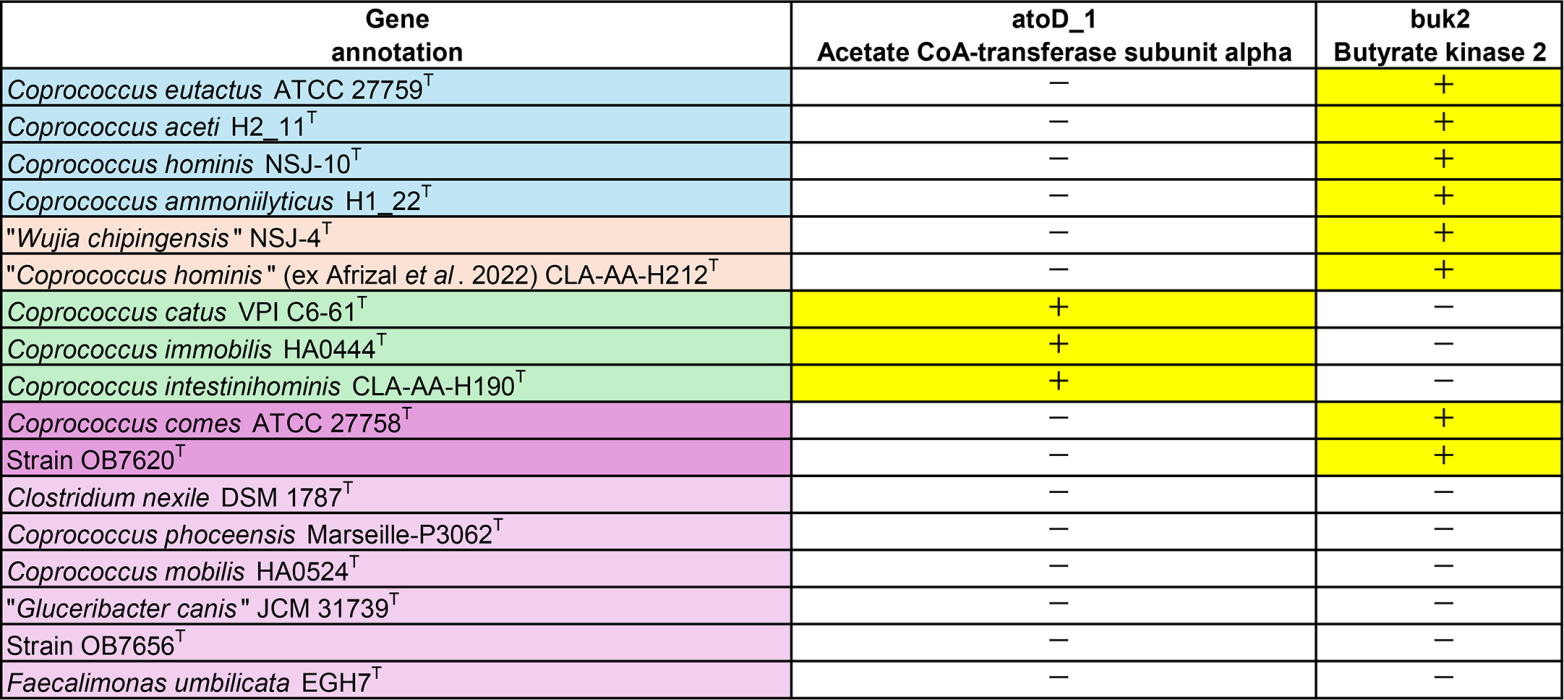
Presence or absence of genes involved in butyrate production. Presence is indicated by + (yellow).

To clarify the systematics of the genus, we conducted a comparative genomic analysis (Fig. 6). We used an automated annotation pipeline based on Prokka (v1.13) [35] to annotate the complete genome sets. Differences in genes between groups were determined using Panaroo (v 1.1.2) [36], with the following options: ‘--mode strict, --core_threshold CORE (=0.50)’. Comparing the genes involved in butyrate synthesis [37] showed that the *buk* gene was present in groups (I), (II) and (IV), whereas the *atoD* gene was only found in group (III). Group (V) lacked both *buk* and *atoD* genes. These findings are consistent with the results for the metabolic end products. Butyrate synthesis genes are useful molecular markers for resolving genus-level relationships among these bacterial taxa.

Fatty acid methyl esters were obtained from ~40 mg of wet cells grown on EG agar for 3 days at 37 °C by saponification, methylation and extraction using minor modifications [38] of the method described by Miller [39]. Cellular fatty acid profiles were determined using version 6.2B of the Sherlock Microbial Identification System (MIDI) and version 3.80 of the BHIBLA database. The major cellular fatty acids (>10%) of strain OB7620^T^ were C_14:0_ (17.1%) and C_16:0_ (19.4%) (Table S3). For strain OB7656^T^, the predominant fatty acids were C_16:0_ (21.0%) and C_18:1_* ω*9*c* dimethyl acetal (DMA) (13.7%).

Based on the results presented here, *C. catus* is placed in a novel genus, *Pseudocoprococcus*, as *Pseudocoprococcus catus* gen. nov., comb. nov. We also conclude that members of the same species, *C. immobilis* and *C. intestinihominis*, are members of the genus *Pseudocoprococcus*. Therefore, we propose the name *Pseudocoprococcus immobilis* gen. nov., comb. nov. Genomic analysis clarified the relationship between *C. comes* and *Coprococcus sensu stricto. C. comes* is transferred to a novel genus, *Allocoprococcus*, as *Allocoprococcus comes* gen. nov., comb. nov. As strain OB7620^T^ is closely related to *C. comes* but is a different species, we propose the name *Allocoprococcus similis* gen. nov., sp. nov. We also conclude that members of the same species, *C. nexile* and *C. phoceensis,* belong to the genus *Faecalimonas*. Therefore, we propose the name *Faecalimonas nexilis* comb. nov. *C. mobilis* and ‘*G. canis*’ also belong to the genus *Faecalimonas*. Therefore, we propose the names *Faecalimonas mobilis* comb. nov. and *Faecalimonas canis* sp. nov. As strain OB7656^T^ is related to ‘*G. canis*’ but is a different species, we propose the name *Faecalimonas hominis* sp. nov. The differential characteristics of strains OB7620^T^, OB7656^T^ and their related taxa are shown in Table S1.

## Description of *Allocoprococcus* gen. nov.

*Allocoprococcus* (Al.lo.co.pro.coc’cus. Gr. masc. pron. *allos*, other; N.L. masc. n. *Coprococcus*, a bacterial genus name; N.L. masc. n. *Allocoprococcus*, another *Coprococcus*).

Cells are Gram-stain-positive, obligately anaerobic, non-spore-forming, non-motile, non-pigmented and coccobacilli found in pairs or chains. Saccharolytic. The end products are acetate, butyrate, formate, lactate and propionate, with small amounts of pyruvate. The predominant cellular fatty acids are C_14:0_ and C_16:0_. This genus belongs to the family *Lachnospiraceae*. The type species is *A. comes*.

## Description of *Allocoprococcus comes* comb. nov.

*Allocoprococcus comes* (co’mes. L. masc./fem. n. *comes*, companion, fellow traveller, referring to the presence of the species in human faeces).

Basonym: *Coprococcus comes* Holdeman and Moore 1974 (Approved Lists 1980 [20]).

The description is as given for *Coprococcus comes* Holdeman and Moore 1974 [17].

The predominant cellular fatty acids are C_14:0_, C_16:0_, C_18:1_* ω*9*c* DMA and C_18:1_* ω*7*c* DMA.

The type strain is VPI C1-38^T^ (=ATCC 27758^T^=JCM 37939^T^), isolated from human faeces. The DNA G+C content of the type strain is 42.5 mol%. The GenBank/EMBL/DDBJ accession numbers for the 16S rRNA gene sequence and whole-genome sequence of ATCC 27758^T^ are EF031542 and CP102277, respectively.

## Description of *Allocoprococcus similis* sp. nov.

*Allocoprococcus similis* (si’mi.lis. L. masc. adj. *similis*, similar, as the type strain is similar to closely related species).

Exhibits the following characteristics, in addition to those given in the description of the genus. Cells are 1.3–1.5×1.8–2.3 µm in size and occur in pairs or chains. Grows at 30–45 °C (optimum, 37 °C) and at pH 5.5–8.0 (optimum, pH 7.0). Growth occurs in the presence of 2% Oxgall (w/v). Colonies on EG agar after 3 days of incubation at 37 °C under anaerobic conditions are 0.7–1.2 mm in diameter, grey, circular, conical and smooth. Aesculin is not hydrolysed. Indole is not produced. Catalase and urease are not produced. Acid is produced from l-arabinose, fructose, galactose, d-glucose, lactose, maltose, d-mannitol, melibiose, raffinose, l-rhamnose, ribose, sucrose and d-xylose, but not from cellobiose, inulin, inositol, d-mannose, melezitose and trehalose. Positive reactions are obtained using the API ZYM system for acid phosphatase, esterase (weak), esterase lipase (weak), α-galactosidase, β-galactosidase, α-glucosidase, β-glucosidase, leucine arylamidase and naphthol-AS-BI-phosphohydrolase. Negative reactions for alkaline phosphatase, *N*-acetyl-β-glucosaminidase, chymotrypsin, cystine arylamidase, α-fucosidase, β-glucuronidase, lipase, α-mannosidase, trypsin and valine arylamidase. Positive reactions are also obtained using Rapid ID 32A for α-galactosidase, β-galactosidase, α-glucosidase, arginine arylamidase, glycine arylamidase, histidine arylamidase, leucyl-glycine arylamidase and pyroglutamic acid arylamidase. Negative reactions for alkaline phosphatase, *N*-acetyl-β-glucosaminidase, α-arabinosidase, arginine dihydrolase, α-fucosidase, β-glucosidase, 6-phospho-β-galactosidase, β-glucuronidase, glutamic acid decarboxylase, alanine arylamidase, glutamyl glutamic acid arylamidase, leucine arylamidase, phenylalanine arylamidase, proline arylamidase, serine arylamidase, tyrosine arylamidase, indole production, nitrate reduction and urease. Mannose and raffinose are not fermented. Predominant cellular fatty acids are C_14:0_ and C_16:0_.

The type strain is OB7620^T^ (=DSM 118890^T^=JCM 37173^T^), isolated from human faeces. The DNA G+C content of the type strain is 42.6 mol%. The GenBank/EMBL/DDBJ accession numbers for the 16S rRNA gene sequence and the whole-genome sequence of OB7620^T^ are LC878528 and BAAHSG010000001–BAAHSG010000078, respectively.

## Description of *Pseudocoprococcus* gen. nov.

*Pseudocoprococcus* (Pseu.do.co.pro.coc’cus. Gr. masc. adj. *pseudes*, false; N.L. masc. n. *Coprococcus*, a bacterial genus name; N.L. masc. n. *Pseudocoprococcus*, false *Coprococcus*).

Cells are Gram-stain-positive, obligately anaerobic, non-spore-forming, non-motile, non-pigmented, elongate cocci found as in pairs or long chains. Saccharolytic. The end products are acetate, butyrate and propionate, with small amounts of pyruvate. The predominant cellular fatty acids are C_18:0_ DMA and C_18:1_* ω*9*c*. This genus is a member of the family *Lachnospiraceae*. The type species is *P. catus*.

## Description of *Pseudocoprococcus catus* comb. nov.

*Pseudocoprococcus catus* (ca’tus. L. masc. adj. *catus*, clever, referring to the unusual property of producing large quantities of both propionate and butyrate).

Basonym: *Coprococcus catus* Holdeman and Moore 1974 (Approved Lists 1980 [20]).

The description is as given for *Coprococcus catus* Holdeman and Moore 1974 [17].

The predominant cellular fatty acids are C_18:0_ DMA, C_18:1_* ω*9*c* and C_18:1_* ω*9*c* DMA.

The type strain is VPI C6-61^T^ (=ATCC 27761^T^=JCM 37941^T^), isolated from human faeces. The DNA G+C content of the type strain is 42.9 mol%. The GenBank/EMBL/DDBJ accession numbers for the 16S rRNA gene sequence and whole-genome sequence of VPI C6-61^T^ are AB038359 and JAAXCM000000000, respectively.

## Description of *Pseudocoprococcus immobilis* comb. nov.

*Pseudocoprococcus immobilis* (im.mo’bi.lis. L. masc. adj. *immobilis*, non-motile, indicating the non-motility of the type strain).

Basonym: *Coprococcus immobilis* Huang *et al*. 2025.

The description is as given for *Coprococcus immobilis* [23], with the following modifications.

The predominant cellular fatty acids are C_16:0_, C_18:0_, C_18:0_ DMA and C_18:1_* ω*9*c* DMA.

The type strain is HA0444^T^ (=JCM 37951^T^=KCTC 25745^T^), isolated from the faeces of patients with rheumatoid arthritis. One additional strain, CLA-AA-H190 (=DSM 14688=LMG 33015), is included in this species. The DNA G+C content of the type strain is 42.9 mol%. The GenBank/EMBL/DDBJ accession numbers for the 16S rRNA gene sequence and whole-genome sequence of JCM 37951^T^ are LC886103 and BAAIAI000000000, respectively.

## Emended description of the genus *Faecalimonas* Sakamoto *et al*. 2017

The description is provided by Sakamoto *et al*. [31], with the following modifications. Cells are non-motile, with the exception of *F. mobilis* ([23] and this study). Spores have only been observed in the type strain of *F. nexilis* [17,40].

## Description of *Faecalimonas canis* sp. nov.

*Faecalimonas canis* (ca’nis. L. gen. n. *canis*, of a dog).

The description is as given for ‘*Gluceribacter canis*’ [40,41].

The predominant cellular fatty acids are C_16:0_ and C_18:1_* *ω9*c* DMA.

The type strain is NATH-2371^T^ (=DSM 105698^T^=JCM 31739^T^), isolated from the faeces of a healthy dog (dachshund, female, 2 years old) in Japan. The DNA G+C content of the type strain is 36.8 mol%. The GenBank/EMBL/DDBJ accession numbers for the 16S rRNA gene sequence of NATH-2371^T^ and the whole-genome sequence of JCM 31739^T^ are LC191811 and BAAHSI010000001–BAAHSI010000054, respectively.

## Description of *Faecalimonas hominis* sp. nov.

*Faecalimonas hominis* (ho’mi.nis. L. gen. n. *hominis*, of a human being, referring to the human gut habitat).

Exhibits the following characteristics, in addition to those given in the description of the genus. Cells are elongated cocci, 0.9–1.1×2.0–2.2 µm in size, and occur in pairs and long chains. Grows at 25–45 °C (optimum, 37 °C) and at pH 5.5–8.5 (optimum, pH 7.0). Growth occurs in the presence of 2% Oxgall (w/v). Colonies on EG agar after 3 days of incubation at 37 °C under anaerobic conditions are 1.4–2.4 mm in diameter, white, opaque, circular, convex and smooth. Aesculin is not hydrolysed. Indole is not produced. Catalase and urease are not produced. Acid is produced from galactose, d-glucose and lactose, but not from l-arabinose, cellobiose, fructose, inulin, inositol, maltose, d-mannitol, d-mannose, melezitose, melibiose, raffinose, l-rhamnose, ribose, sucrose, trehalose and d-xylose. Positive reactions are obtained using the API ZYM system for *N*-acetyl-β-glucosaminidase, acid phosphatase, esterase (weak), esterase lipase (weak), β-galactosidase and naphthol-AS-BI-phosphohydrolase. Negative reactions are obtained for alkaline phosphatase, chymotrypsin, cystine arylamidase, α-fucosidase, α-galactosidase, α-glucosidase, β-glucosidase, β-glucuronidase, leucine arylamidase, lipase, α-mannosidase, trypsin and valine arylamidase. Positive reactions are also obtained using Rapid ID 32A for *N*-acetyl-β-glucosaminidase, β-galactosidase, proline arylamidase and pyroglutamic acid arylamidase. Negative reactions are obtained for alkaline phosphatase, α-arabinosidase, arginine dihydrolase, α-fucosidase, α-galactosidase, 6-phospho-β-galactosidase, α-glucosidase, β-glucosidase, β-glucuronidase, glutamic acid decarboxylase, alanine arylamidase, arginine arylamidase, glutamyl glutamic acid arylamidase, glycine arylamidase, histidine arylamidase, leucine arylamidase, leucyl-glycine arylamidase, phenylalanine arylamidase, serine arylamidase, tyrosine arylamidase, indole production, nitrate reduction and urease. Mannose and raffinose are not fermented. Predominant cellular fatty acids are C_16:0_ and C_18:1_* ω*9*c* DMA.

The type strain is OB7656^T^ (=DSM 118889^T^=JCM 37172^T^), isolated from human faeces. The DNA G+C content of the type strain is 38.9 mol%. The GenBank/EMBL/DDBJ accession numbers for the 16S rRNA gene sequence and the whole-genome sequence of OB7656^T^ are LC878529 and BAAHSH010000001–BAAHSH010000099, respectively.

## Description of *Faecalimonas mobilis* comb. nov.

*Faecalimonas mobilis* (mo’bi.lis. L. fem. adj. *mobilis*, motile).

Basonym: *Coprococcus mobilis* Huang *et al*. 2025.

The description is given for *Coprococcus mobilis* [23].

The predominant cellular fatty acids are C_14:0_, C_16:0_, C_16:0_ DMA and C_18:1_* ω*9*c* DMA.

The type strain is HA0524^T^ (=CGMCC 1.48247^T^=JCM 37798^T^), isolated from the faeces of patients with rheumatoid arthritis. The DNA G+C content of the type strain is 40.0 mol%. The GenBank/EMBL/DDBJ accession numbers for the 16S rRNA gene sequence and the whole-genome sequence of JCM 37798^T^ are LC886102 and BAAIAH000000000, respectively.

## Description of *Faecalimonas nexilis* comb. nov.

*Faecalimonas nexilis* (ne’xi.lis. L. fem. adj. *nexilis*, tied or bound together, referring to its chain formation).

Basonym: *Clostridium nexile* Holdeman and Moore 1974 (Approved Lists 1980 [20]).

The description is as given for *Clostridium nexile* Holdeman and Moore 1974 [17].

The predominant cellular fatty acids are C_14:0_, C_16:0_ and C_18:1_* ω*9*c* DMA.

The type strain is VPI C48-37^T^ (=ATCC 27757^T^=DSM 1787^T^=JCM 31500^T^), isolated from human faeces. One additional strain, Marseille-P3062 (=DSM 103635=JCM 37525), is included in this species. The DNA G+C content of the type strain is 40.1 mol%. The GenBank/EMBL/DDBJ accession numbers for the 16S rRNA gene sequence of JCM 31500^T^ and the whole-genome sequence of DSM 1787^T^ are LC519856 and ABWO00000000, respectively.

## Emended description of *Coprococcus ammoniilyticus* Hitch *et al*. 2022

The description is given by Hitch *et al*. [13] with the following modifications. The predominant cellular fatty acid is C_16:0_. One additional strain, NSJ-10 (=CGMCC 1.32463=JCM 37066), is included in this species.

## Supplementary material

10.1099/ijsem.0.007011 Uncited Supplementary Material 1.

## Abbreviations

AAI: average amino acid identity
ANI: average nucleotide identity
ATCC: American Type Culture Collection
dDDH: digital DNA–DNA hybridization
GAM: Gifu anaerobic medium
GGDC: Genome-to-Genome Distance Calculator
GTDB: Genome Taxonomy Database
JCM: Japan Collection of Microorganisms
MIDI: Microbial Identification System
ML: maximum-likelihood
POCP: percentage of conserved proteins
SEM: scanning electron microscopy
