# Supplementary material for: Proposal of two novel species, Allocoprococcus similis gen. nov., sp. nov. and Faecalimonas hominis sp. nov., isolated from human faeces and genome-based reorganization of the genus Coprococcus
Source: Int J Syst Evol Microbiol. 2026 Jan 7;76(1):007011. doi: 10.1099/ijsem.0.007011 (PMC12778736; doi:10.1099/ijsem.0.007011)
Supplement: Uncited Supplementary Material 1. [file ijsem-76-07011-s001.pdf]

Proposal of two novel species, *Allocoprococcus similis* gen. nov., sp. nov. and *Faecalimonas hominis* sp. nov., isolated from human faeces and genome-based reorganisation of the genus *Coprococcus*

Eri Yamamoto[1,*,†], Atsushi Hisatomi[2,†], Kana Miwa[2], Naomi Sakurai[2], Akiko Koizumi[1], Moriya Ohkuma[2], Hanae Tsuchihashi[1] and Mitsuo Sakamoto[2,3,*]

Author affiliations: [1]Microbial Bioresources Group, Lactic Acid Bacteria & Fermentation Technology Research Unit, R&D Division, Meiji Co., Ltd., Hachioji, Tokyo 192-0919, Japan; [2]Microbe Division/Japan Collection of Microorganisms, RIKEN BioResource Research Center, Tsukuba, Ibaraki 305-0074, Japan; [3]NODAI Culture Collection Center, Tokyo NODAI Research Institute, Tokyo University of Agriculture, Setagaya-ku, Tokyo 156-8502, Japan.

***Correspondence:** Eri Yamamoto, eri.yamamoto@meiji.com; Mitsuo Sakamoto, sakamoto@riken.jp

†These authors contributed equally to this work and should be regarded as co-first authors.

Table S1. Differential characteristics of strains OB7620^{T}, OB7656^{T}, and related taxa

Strains: 1, *C. eutactus* JCM 37940^{T} (=ATCC 27759^{T}); 2, *C. aceti* JCM 31265^{T}; 3, *C. hominis* JCM 37066^{T}; 4, *C. ammoniilyticus* JCM 34806^{T}; 5, '*W. chipingensis*' NSJ-4^{T}; 6, '*C. hominis*' CLA-AA-H212^{T} (*ex* Afrizal *et al*. 2022); 7, *C. catus* JCM 37941^{T} (=ATCC 27761^{T}); 8, *C. immobilis* JCM 37951^{T}; 9, *C. intestinihominis* CLA-AA-H190^{T}; 10, *C. comes* JCM 37939^{T} (=ATCC 27758^{T}); 11, strain OB7620^{T} (=JCM 37173^{T}); 12, *Cl. nexile* JCM 31500^{T}; 13, *C. phoceensis* JCM 37525^{T}; 14, *C. mobilis* JCM 37798^{T}; 15, '*G. canis*' JCM 31739^{T}; 16, strain OB7656^{T} (=JCM 37172^{T}); 17, *F. umbilicata* JCM 30896^{T}. Data were obtained from [2, 15, 22] and this study. +, positive; -, negative; w, weak; ND, no data available.

	Coprococcus				**'*Wujia*'**		***Pseudocoprococcus***			***Allocoprococcus***		***Faecalimonas***					
**Characteristic**	**1**	**2**	**3**	**4**	**5**	**6**	**7**	**8**	**9**	**10**	**11**	**12**	**13**	**14**	**15**	**16**	**17**
End product of metabolism*	A, B, F, L, Pr, Py	A, B, F, L, Pr	A, B, F, L, Pr, Py	A, B, F, L, Pr	ND	A, Pr	A, B, Pr, Py	A, B, F, L, Pr	A, B, Pr	A, B, F, L, Pr	A, B, F, L, Pr, Py	A, F, L, Pr, Py	A, F, L, Pr, Py	A, F, L, Pr, Py	A, F, L, Pr, Py	A, F, L, Pr, Py	A, F, L, Pr, Py
Motility	-	-	-	-	+ (flagella)	+ (annotation)	-	-	ND	-	-	-	-	+ (flagella)	-	-	-
Growth in bile	+	+	+	-	ND	ND	+	+	ND	+	+	+	+	+	+	+	+
Spore formation	-	-	-	-	ND	ND	-	-	ND	-	-	+	-	-	-	-	-
H_2S production	-	-	-	+	ND	ND	-	-	ND	-	+	w	-	-	-	-	+
Esculin hydrolysis	w	w	+	+	ND	ND	-	-	ND	-	-	+	w	+	-	-	+
Fermentation of																	
Arabinose	-	-	-	-	ND	ND	-	-	ND	+	+	-	-	-	-	-	-
Cellobiose	+	+	+	+	ND	ND	-	-	ND	-	-	+	-	-	-	-	-
Fructose	+	+	+	+	ND	ND	+	+	ND	+	+	+	+	+	+	-	+
Inulin	-	-	-	-	ND	ND	-	-	ND	+	-	-	-	-	-	-	+
Lactose	+	+	+	+	ND	ND	-	-	ND	+	+	+	+	+	+	+	+
Mannose	+	-	-	-	ND	ND	-	-	ND	-	-	-	-	-	-	-	-
Maltose	+	+	+	+	ND	ND	-	-	ND	+	+	-	-	+	-	-	-
Mannitol	-	-	-	-	ND	ND	+	+	ND	+	+	-	-	-	-	-	-
Melezitose	+	+	-	-	ND	ND	-	-	ND	-	-	-	-	-	-	-	-
Raffinose	+	+	+	+	ND	ND	-	-	ND	+	+	+	+	-	-	-	+
Ribose	-	-	w	+	ND	ND	-	-	ND	-	+	-	-	-	-	-	+
Sucrose	+	+	w	+	ND	ND	-	-	ND	+	+	+	+	+	+	-	-
Xylose	-	-	-	-	ND	ND	-	-	ND	+	+	+	+	-	-	-	+
Major fatty acids (>10%)	$C_{16:0}$, $C_{14:0}$ DMA, iso-$C_{13:0}$ 3OH	$C_{16:0}$, iso-$C_{13:0}$ 3OH	$C_{16:0}$	$C_{16:0}$	ND	ND	$C_{18:0}$ DMA, $C_{18:1}$ *ω9c*, $C_{18:1}$ *ω9c* DMA	$C_{16:0}$, $C_{18:0}$, $C_{18:0}$ DMA, $C_{18:1}$ *ω9c*	ND	$C_{14:0}$, $C_{16:0}$, $C_{18:1}$ *ω9c* DMA, $C_{18:1}$ *ω7c* DMA	$C_{14:0}$, $C_{16:0}$	$C_{14:0}$, $C_{16:0}$, $C_{18:1}$ *ω9c* DMA	$C_{16:0}$, $C_{18:1}$ *ω9c* DMA, $C_{18:1}$ *ω7c* DMA	$C_{14:0}$, $C_{16:0}$, $C_{16:0}$ DMA, $C_{18:1}$ *ω9c* DMA	$C_{16:0}$, $C_{18:1}$ *ω9c* DMA	$C_{16:0}$, $C_{18:1}$ *ω9c* DMA	$C_{16:0}$, $C_{18:1}$ *ω9c* DMA, $C_{18:1}$ *ω7c* DMA
DNA G+C content (%)	43.1	43.7	41.2	41.0	43.9	44.2	43.0	42.9	43.3	42.5	42.6	40.1	40.2	40.0	36.8	38.9	41.5
Source	Human faeces	Human faeces	Human faeces	Human faeces	Human faeces	Human faeces	Human faeces	Human faeces	Human faeces	Human faeces	Human faeces	Human faeces	Human left colon	Human faeces	Dog faeces	Human faeces	Human faeces

*A, acetate; B, butyrate; F, formate; L, lactate; Pr, propionate; Py, pyruvate.

Table S2. Fermentation products (mM) formed by strains tested

Strains: 1, *C. eutactus* JCM 37940^{T} (=ATCC 27759^{T}); 2, *C. aceti* JCM 31265^{T}; 3, *C. hominis* JCM 37066^{T}; 4, *C. ammoniilyticus* JCM 34806^{T}; 5, *C. catus* JCM 37941^{T} (=ATCC 27761^{T}); 6, *C. immobilis* JCM 37951^{T}; 7, *C. comes* JCM 37939^{T} (=ATCC 27758^{T}); 8, strain OB7620^{T} (=JCM 37173^{T}); 9, *Cl. nexile* JCM 31500^{T}; 10, *C. phoceensis* JCM 37525^{T}; 11, *C. mobilis* JCM 37798^{T}; 12, '*G. canis*' JCM 31739^{T}; 13, strain OB7656^{T} (=JCM 37172^{T}); 14, *F. umbilicata* JCM 30896^{T}.

**Strain**	**Acetate**	**Butyrate**	**Formate**	**Lactate**	**Propionate**	**Pyruvate**
1	3.61 ± 0.27	7.29 ± 0.19	18.47 ± 0.59	8.08 ± 1.22	0.11 ± 0.07	0.10 ± 0.02
2	2.07 ± 0.20	2.69 ± 0.46	7.62 ± 1.02	28.87 ± 1.73	0.08 ± 0.08	-
3	2.98 ± 0.12	7.64 ± 0.15	18.1 ± 0.32	10.43 ± 0.94	0.11 ± 0.08	0.29 ± 0.00
4	2.45 ± 0.04	4.36 ± 0.19	11.28 ± 0.39	21.29 ± 1.67	0.12 ± 0.08	-
5	2.58 ± 0.45	2.73 ± 0.59	-	-	2.85 ± 0.24	0.05 ± 0.00
6	2.50 ± 1.15	4.31 ± 3.47	1.84 ± 0.13	5.60 ± 0.88	5.95 ± 0.37	-
7	8.47 ± 0.74	22.29 ± 6.48	2.55 ± 1.14	29.08 ± 6.14	2.69 ± 0.09	-
8	6.73 ± 0.56	19.61 ± 2.12	2.77 ± 0.24	38.36 ± 2.07	3.19 ± 0.04	0.08 ± 0.03
9	6.99 ± 3.39	-	5.45 ± 3.23	7.39 ± 0.44	5.24 ± 0.32	0.02 ± 0.01
10	9.35 ± 3.61	-	6.39 ± 2.67	7.96 ± 0.51	5.52 ± 0.22	0.08 ± 0.04
11	4.26 ± 0.83	-	2.27 ± 0.25	6.31 ± 0.38	5.11 ± 0.30	0.04 ± 0.02
12	9.36 ± 0.99	-	17.16 ± 2.13	0.07 ± 0.01	2.19 ± 0.06	0.88 ± 0.76
13	16.74 ± 0.46	-	18.71 ± 0.54	0.35 ± 0.05	1.4 ± 0.09	1.77 ± 1.26
14	16.49 ± 2.57	-	18.21 ± 2.55	0.54 ± 0.21	0.12 ± 0.10	0.95 ± 0.80

Table S3. Cellular fatty acid compositions of strains OB7620T, OB7656T, and related taxa

Strains: 1, *C. eutactus* JCM 37940^T (=ATCC 27759^T); 2, *C. aceti* JCM 31265^T; 3, *C. hominis* JCM 37066^T; 4, *C. ammoniilyticus* JCM 34806^T; 5, *C. catus* JCM 37941^T (=ATCC 27761^T); 6, *C. immobilis* JCM 37951^T; 7, *C. comes* JCM 37939^T (=ATCC 27758^T); 8, strain OB7620^T (=JCM 37173^T); 9, *Cl. nexile* JCM 31500^T; 10, *C. phoceensis* JCM 37525^T; 11, *C. mobilis* JCM 37798^T; 12, '*G. canis*' JCM 31739^T; 13, strain OB7656^T (=JCM 37172^T); 14, *F. umbilicata* JCM 30896^T. Each value represents the mean of three experiments. Major components (>10 %) are highlighted in bold.

Fatty acid	1	2	3	4	5	6	7	8	9	10	11	12	13	14
$C_{9:0}$		0.2	0.2		0.3									
$C_{10:0}$			0.2		0.1			0.1	0.2		0.2			0.1
$C_{12:0}$	0.7	1.3	0.6	0.6	0.9	1.4	0.7	1.1	3.0	1.7	4.8	2.4	2.0	0.9
$C_{13:0}$							0.1		0.2	0.2	0.3	0.2	0.2	0.1
$C_{14:0}$	8.9	7.0	8.0	4.3	1.9	2.2	**15.0**	**17.1**	**10.4**	6.2	**11.7**	8.6	8.5	2.4
$C_{15:0}$	0.4	0.2	0.2	0.1	0.3		0.1	0.1	0.3	0.3	0.3	0.2	0.2	0.1
$C_{16:0}$	**37.4**	**38.3**	**44.4**	**44.7**	9.2	**11.5**	**14.7**	**19.4**	**18.2**	**17.5**	**16.1**	**19.9**	**21.0**	**17.4**
$C_{17:0}$	0.3	0.2	0.3	0.5										
$C_{18:0}$	2.2	2.5	2.8	3.4	8.6	**10.0**	1.7	0.8	0.8	0.9	0.8	1.4	1.7	6.4
$C_{16:0}$ 2OH					2.3	2.9								
$C_{16:0}$ 3OH	0.5	0.4	0.2				0.1			0.1		0.1	0.2	0.2
$C_{11:0}$ DMA	0.4	0.5		0.1	0.3	1.2	0.5	0.7	0.5	0.4	1.4	0.3	0.7	0.2
$C_{14:0}$ DMA	**10.2**	8.5	8.0	4.7	1.5	2.2	4.1	5.4	1.7	1.2	3.4	1.8	2.5	0.4
$C_{16:0}$ DMA	2.6	1.8	5.9	8.9	3.8	8.2	4.5	3.5	6.0	6.0	**11.3**	8.5	8.5	5.6
$C_{17:0}$ DMA	0.2	0.2								0.1		0.1		0.1
$C_{18:0}$ DMA			0.2	0.3	**19.5**	**14.0**	1.2	0.4	0.9	1.4	1.5	1.0	1.0	4.1
$C_{16:0}$ ALDE	0.8	0.8	1.3	1.9	0.7	3.1	0.8	0.7	1.7	1.8	3.7	1.6	1.4	1.1
$C_{18:0}$ ALDE					3.6	4.6	0.2	0.1	0.3	0.5	0.4	0.3	0.3	0.9
$C_{22:0}$ NHC	0.7			0.8	0.5									
iso-$C_{12:0}$	0.1													
iso-$C_{14:0}$	2.0	3.9	4.5	3.3										
iso-$C_{16:0}$	1.8	3.1	1.7	2.1										
iso-$C_{13:0}$ 3OH	**14.6**	**15.3**	9.4	8.9										
anteiso-$C_{15:0}$	0.2	0.4	0.5	0.4										

anteiso-$C_{15:0}$ DMA	1.7	1.9	1.3	1.4										
anteiso-$C_{17:0}$	0.2							0.2	0.3	0.7	0.5	0.2	0.1	0.2
$C_{14:1}\omega 5c$									0.2	0.1				
$C_{16:1}\omega 9c$							0.4	0.3	1.0	0.7	0.6	0.3	0.4	0.1
$C_{16:1}\omega 7c$	0.1				0.3		3.6	3.3	2.6	1.3	1.5	2.2	2.2	0.7
$C_{16:1}\omega 5c$							0.4	0.4	0.4	0.4		0.1	0.1	0.3
$C_{18:1}\omega 9c$	3.0	3.3	2.6	3.1	**13.8**	**13.5**	5.7	5.5	7.9	6.0	4.0	6.8	9.0	6.7
$C_{18:1}\omega 5c$										0.3		0.1	0.1	0.7
$C_{20:1}\omega 9c$												0.1	0.1	0.1
$C_{14:1}\omega 7c$ DMA							0.9	0.9		0.1	0.4	0.1	0.1	
$C_{16:1}\omega 7c$ DMA							8.0	7.2	3.2	2.5	2.8	4.1	3.7	1.0
$C_{18:1}\omega 9c$ DMA	0.6	0.3	0.2	0.2	**11.5**	9.4	**11.3**	9.7	**13.4**	**16.4**	**10.9**	**16.3**	**13.7**	**15.6**
$C_{18:1}\omega 7c$ DMA	0.4	0.6	0.3	0.5	6.9	5.5	**10.1**	9.1	8.7	**13.1**	6.9	9.6	8.1	**16.1**
$C_{18:2}\omega 9,6c$	0.8	1.1	1.0	0.9	4.4	7.4	0.7	0.8	3.0	2.7	1.8	1.1	1.3	1.0
$C_{15:1}$ $\omega 9$/t7c	0.2			0.3				0.1		0.1				0.1
$C_{18:1}$ AT 17.254 DMA										0.1		0.1	0.1	0.1
$C_{17:0}$ CYC DMA													0.1	
$C_{19:0}$ CYC 11.12 DMA														0.2
$C_{20:1}$ c13/t11													0.1	0.3
unknown 16.107 16i DMA	1.0	1.3	2.0	3.9										
Summed Feature 1 ($C_{13:1}$ ω 1 and/or $C_{14:0}$ ALDE)	4.9	5.2	3.1	1.9	0.5	1.6	1.2	1.3	0.5	0.4	1.3	0.6	0.7	0.2
Summed Feature 2 ($C_{12:0}$ 3OH and/or $C_{13:0}$ DMA)									0.1		0.2		0.1	
Summed Feature 4 (unknown 14.762 and/or $C_{15:2}$)							2.0	1.7	1.3	1.3	1.5	1.5	1.0	0.4
Summed Feature 5 ($C_{15:0}$ DMA and/or $C_{14:0}$ 3OH)	0.1						0.3	0.1	0.2	0.1	0.4	0.3	0.3	0.2
Summed Feature 6 (anteiso-$C_{15:0}$ 3OH and/or $C_{16:1}$ $\omega 9c$ DMA)							0.4	0.4	1.5	1.3	1.6	1.4	1.3	0.3
Summed Feature 7 ($C_{17:2}$ and/or $C_{17:1}$ $\omega 9c$)	0.3				1.9	3.5	1.7	1.9	3.8	4.9	3.2	2.8	2.1	2.5
Summed Feature 8 ($C_{17:1}$ $\omega 8c$ and/or $C_{17:2}$)					1.9	2.0	1.5	1.9	2.7	4.1	2.1	1.6	1.3	2.5
Summed Feature 10 ($C_{18:1}$ c11/t9/t6 and/or unknown 17.834)	4.2	3.2	2.2	4.0	**10.7**	6.3	8.2	5.4	4.9	5.0	3.2	4.4	5.9	**11.2**
Summed Feature 11 (iso-$C_{17:0}$ 3OH and/or $C_{18:2}$ DMA)	0.2	0.1						0.4	0.8	1.1	1.1	0.5	0.4	0.5
Summed Feature 12 (unknown 18.622 and/or iso-$C_{19:0}$)							0.1							0.2
